# Dual-Model Radiomic Biomarkers Predict Development of Mild Cognitive Impairment Progression to Alzheimer’s Disease

**DOI:** 10.3389/fnins.2018.01045

**Published:** 2019-01-11

**Authors:** Hucheng Zhou, Jiehui Jiang, Jiaying Lu, Min Wang, Huiwei Zhang, Chuantao Zuo, Alzheimer’s Disease Neuroimaging Initiative

**Affiliations:** ^1^Shanghai Institute for Advanced Communication and Data Science, Shanghai University, Shanghai, China; ^2^PET Center, Huashan Hospital, Fudan University, Shanghai, China; ^3^Institute of Functional and Molecular Medical Imaging, Fudan University, Shanghai, China; ^4^Human Phenome Institute, Fudan University, Shanghai, China

**Keywords:** Alzheimer’s disease, mild cognitive impairment, radiomics, image fusion, Cox model

## Abstract

Predicting progression of mild cognitive impairment (MCI) to Alzheimer’s disease (AD) is clinically important. In this study, we propose a dual-model radiomic analysis with multivariate Cox proportional hazards regression models to investigate promising risk factors associated with MCI conversion to AD. T1 structural magnetic resonance imaging (MRI) and ^18^F-Fluorodeoxyglucose (FDG) positron emission tomography (PET) data, from the AD Neuroimaging Initiative database, were collected from 131 patients with MCI who converted to AD within 3 years and 132 patients with MCI without conversion within 3 years. These subjects were randomly partition into 70% training dataset and 30% test dataset with multiple times. We fused MRI and PET images by wavelet method. In a subset of subjects, a group comparison was performed using a two-sample *t*-test to determine regions of interest (ROIs) associated with MCI conversion. 172 radiomic features from ROIs for each individual were established using a published radiomics tool. Finally, L1-penalized Cox model was constructed and Harrell’s C index (C-index) was used to evaluate prediction accuracy of the model. To evaluate the efficacy of our proposed method, we used a same analysis framework to evaluate MRI and PET data separately. We constructed prognostic Cox models with: clinical data, MRI images, PET images, fused MRI/PET images, and clinical variables and fused MRI/PET images in combination. The experimental results showed that captured ROIs significantly associated with conversion to AD, such as gray matter atrophy in the bilateral hippocampus and hypometabolism in the temporoparietal cortex. Imaging model (MRI/PET/fused) provided significant enhancement in prediction of conversion compared to clinical models, especially the fused-modality Cox model. Moreover, the combination of fused-modality imaging and clinical variables resulted in the greatest accuracy of prediction. The average C-index for the clinical/MRI/PET/fused/combined model in the test dataset was 0.69, 0.73, 0.73 and 0.75, and 0.78, respectively. These results suggested that a combination of radiomic analysis and Cox model analyses could be used successfully in survival analysis and may be powerful tools for personalized precision medicine patients with potential to undergo conversion from MCI to AD.

## Introduction

Alzheimer’s disease (AD) is the most common form of progressive, neurodegenerative, and irreversible dementia, with prevalence doubling approximately every 5 years after 65 years of age (Hurd et al., 2013). Since AD may progress for many years before cognitive symptoms appear, and cognitive deficits are evident before the onset of comprehensive dementia syndrome, growing attention has focused on mild cognitive impairment (MCI) as an intermediate state between normal cognition and AD (Brooks and Loewenstein, 2010). Early diagnosis of AD is difficult, with efforts focused on through assessment of the earliest cognitive and neuropathological changes associated with early AD. Significant challenges exist in identifying cases of MCI over time with potential to progress to AD. However, the neuropathological correlates of MCI are heterogeneous (Schneider et al., 2009) and not all cases of MCI progress to dementia. Furthermore, not all cases of dementia will result in a diagnosis of AD (Petersen et al., 2009). Therefore, it is necessary gain greater understanding of the specific risk factors and biomarkers that predict progression from MCI to AD.

Currently, structural and functional neuroimaging are used to predict development of AD from MCI (Devanand et al., 2007; Schneider et al., 2009; Gomar et al., 2011; Caroli et al., 2012; Vos et al., 2012; Prestia et al., 2013b; Richard et al., 2013; Teipel et al., 2013; Frölich et al., 2017; Pagani et al., 2017). Measurement of markers derived from magnetic resonance images (MRI) such as brain morphological changes (Schneider et al., 2009) and reduced hippocampal volumes (Devanand et al., 2007; Gomar et al., 2011; Vos et al., 2012; Prestia et al., 2013b; Teipel et al., 2013) have proven to have predictive value. A previous study used cortical thickness in the right anterior cingulate and middle frontal gyri as predictive features of conversion from MCI to AD, with an accuracy of 75% (Peters et al., 2014). Various studies have shown that MCI patients exhibit metabolic changes that can be detected using ^18^F-fluorodeoxyglucose (FDG) positron emission tomography (PET) (Caroli et al., 2012; Liu et al., 2017; Pagani et al., 2017). Regional variations in glucose metabolism correlate with cognitive impairment (Haense et al., 2008). FDG PET hypometabolism is also associated with progression from pre-MCI to MCI (Caselli et al., 2008) and from amnestic MCI (aMCI) to AD (Anchisi et al., 2005). In addition, FDG PET is the only technique that significantly improved the predictive value of demographic covariates with regard to development of AD (Shaffer et al., 2013; Ito et al., 2015). Specifically, FDG PET accurately predicted progression of MCI to AD in 70 and 83% of cases in two studies (Landau et al., 2010; Chen et al., 2011; Ito et al., 2015). Although these results were statistically significant, these results were not accurate enough to warrant use in clinical diagnosis. To identify MCI patients at imminent risk of conversion, a multimodal approach has been used, resulting in prediction of MCI conversion to AD with relatively high accuracy (Arbizu et al., 2013; Ewers et al., 2014; Moradi et al., 2015; Liu et al., 2017). Medical image fusion was used to combine complementary information provided by different brain imaging technologies to identify biomarkers to aid in MCI recognition and classification based on different aspects of brain changes. Liu et al. (2017) found that combined neuroimaging factors derived from MRI and FDG PET images, together with clinical variables, can effectively predict the time to progression from MCI to AD. Research performed by Dickerson and Wolk (2013) showed that prediction of time-to-event in a survival model was accomplished by combination of the MRI biomarkers of AD cortical thickness and cerebrospinal fluid (CSF). However, the aforementioned markers used in these methods were mostly low-level features incapable of providing accurate diagnostic results due to neuropathological heterogeneity of brain tissue related to conversion of MCI to AD. To improve diagnostic accuracy of prediction of conversion of MCI to AD, radiomics, a recently developed method, may show promise.

Radiomics was recently developed as a diagnostic and auxiliary detection technique for oncological studies (Aerts et al., 2014; Vallières et al., 2015; Cameron et al., 2016; Zhou et al., 2017). This technique is characterized by extraction and analysis of large amounts of advanced and high-order quantitative features with high-throughput from medical images using a large number of automated feature extraction algorithms (Kumar et al., 2012). These radiomic features may help to effectively diagnose disease and reveal in-depth information not readily apparent in standard imaging analyses which may aid in development of personalized and accurate medical plans (Kumar et al., 2012; Gillies et al., 2015; Panth et al., 2015). Radiomics has been applied to various cancers, such as glioma (Li et al., 2018), lung cancer (Aerts et al., 2014; Tang et al., 2018), hepatocellular carcinoma (Cozzi et al., 2017), rectal cancer (Meng et al., 2018), head and neck cancer (Aerts et al., 2014; M. D. Anderson Cancer Center Head and Neck Quantitative Imaging Working Group, 2018), and breast cancer (Cameron et al., 2016). Although most radiomics research studies focused mainly on oncology, this methodology has recently extended to a large number of (Yu et al., 2016) medical applications (Gillies et al., 2015). Recently, radiomics has been used to evaluate other diseases, such as autism spectrum disorders (Chaddad et al., 2017), attention deficit hyperactivity disorder (Sun et al., 2017), and xerostomia (Gabryś et al., 2018). The purpose of this study was to use radiomics to predict conversion from MCI to AD. We believe that advanced radiomic features could fully account for brain tissue heterogeneity in patients with MCI, allowing for identification of patients likely to convert to AD.

The present study aimed to investigate risk factors identified by radiomics analysis and to evaluate their effects on MCI conversion to AD by combining MRI and FDG PET imaging combined with radiomics analysis. Two main goals of this study were to: (1) determine which radiomic features derived from fused MRI and PET images are related to disease progression, lending support to clinical decision-making; (2) compare predictive accuracy of MCI conversion to AD using radiomic features derived from fused MRI and PET images to improve predictive accuracy of conversion using radiomic features derived from only MRI or FDG PET alone.

## Materials and Methods

**FIGURE 1:**
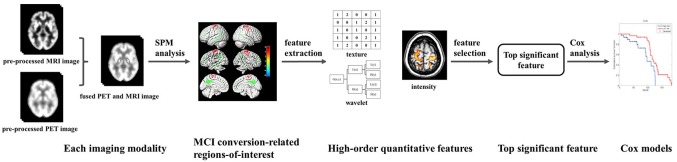
The overall framework of the experimental process in this study.

Figure 1 summarizes the framework of the experimental design in our study. We first preprocessed the collected MRI data (segmentation, normalization, and smoothing) and PET data (normalization and smoothing). We used the image fusion algorithm to fuse the spatial and frequency characteristics of the MRI and FDG PET data. After image fusion, we performed SPM analysis based on a voxel-wise two-sample t-test statistical model of the fused images to determine MCI conversion-related ROIs. Subsequently, a series of radiomic and contrast features were extracted from the ROIs. The top radiomic features associated with MCI conversion were selected. Based on these selected radiomic features, we constructed Cox proportional hazards regression models to examine the diagnostic value of candidate predictors.

### Subjects

The FDG PET and T1-weighted structural MRI scans used in our study were obtained from the Alzheimer’s Disease Neuroimaging Initiative (ADNI) database^1^. ADNI was launched in 2003 by the National Institute on Aging (NIA), the National Institute of Biomedical Imaging and Bioengineering (NIBIB), the Food and Drug Administration (FDA), private pharmaceutical companies, and non-profit organizations, as a $60 million, 5-year public–private partnership. The primary goal of ADNI has been to test whether serial magnetic resonance imaging (MRI), PET, other biological markers, and clinical and neuropsychological assessment can be combined to measure progression of MCI and early AD.

We collected image data from ADNI 1, ADNI 2, and ADNI GO cohorts in the ADNI database using the following inclusion criteria: (1) Participants were diagnosed with MCI at baseline; (2) Participants must have had both MRI and PET scans at the time of data collection; (3) For MCI non-converters, participants were evaluated for at least 3 years (including a 3 years time point) from the time of initial data collection (for MCI converters, the evaluation time may be less than 3 years). Scan data for MCI non-converters were collected at baseline and these participants did not convert to AD during the 3 years follow-up period. Scan data for MCI converters were not all collected at baseline. Since few MCI converters had both MRI and PET scans at baseline, we collected the scan data at time points at which both MRI and PET scans were collected. Participants with a bidirectional change of diagnosis (MCI to AD, and back to MCI) within the follow-up period were excluded.

The MRI and PET scan data were collected for 131 MCI converters (MCI-c) and 132 MCI non-converters (MCI-nc). 73 individuals of the 131 MCI-c had MRI and PET scan data at baseline, and the remaining 58 individuals were non-baseline data, the time between baseline and MR/PET scan data range from 6 to 72 months (median = 18 months, mean = 21.2 months). Scan data for all MCI-nc were collected at baseline. Clinical and demographic characteristics of the data set are shown in Table 1. As expected, the apolipoprotein E (APOE) ε4 positive rate was significantly different for MCI-c versus MCI-nc.

**Table 1:** Demographic and statistics of clinical assessments at time of data collection.

	MCI-c		MCI-nc	
	(*n* = 131)		(*n* = 132)	
		
	Median (IQR)	Mean (*SD*)	Median (IQR)	Mean (*SD*)
Sex (M/F)	78/53		66/66	
Age (years)	74.4 (8.9)	73.8 (6.9)	72.3 (12.3)	72.1 (7.8)
MMSE	27.0 (3.0)	26.7 (2.0)	28.0 (2.0)	28.0 (1.6)
APOE ε4 positive rate	54.2%		31.1%	

	**A subset of all subjects**			

	**MCI-c**		**MCI-nc**	
	**(*n* = 81)**		**(*n* = 82)**	

Sex (M/F)	44/37		36/46	
Age (years)	74.5 (8.4)	73.3 (7.1)	74.8 (12.4)	73.9 (7.8)
MMSE	27.0 (3.0)	27.0 (1.8)	28.0 (2.0)	27.7 (1.7)
APOE ε4 positive rate	56.8%		28.1%	


*MCI-c, MCI converters; MCI-nc, MCI non-converters; IQR, interquartile range; SD, standard deviation*.

### Image Preprocessing

#### ^18^F-Fluorodeoxyglucose (FDG) Positron Emission Tomography (PET)

The PET acquisition process is detailed in the online information of the ADNI project. In 207 cases, dynamic 3D scans with six 5-min frames were acquired 30 min after injection of 185 ± 18.5 MBq FDG. In the remaining cases (*n* = 56), patients were scanned for a static 30-min acquisition period. In the case of dynamic scans, all frames were motion-corrected to the first frame and then summed to create a single image file.

Individual PET scan preprocessing was performed by statistical parametric mapping (SPM12) software (Wellcome Department of Imaging Neuroscience, Institute of Neurology, London, United Kingdom) using Matlab2016b (Mathworks Inc, Sherborn, MA, United States). Scans from each subject were spatially normalized into Montreal Neurological Institute (MNI) space with linear and non-linear 3D transformations. The normalized PET images were then smoothed by a Gaussian filter of 8 mm full-width at half-maximum (FWHM) over a 3D space to blur the individual anatomical variations and to increase the signal-to-noise ratio for subsequent analysis. Finally, given that the difference in the FDG uptake value of each individual, the smoothed image was normalized to the range of 0 to 255.

#### Magnetic Resonance Imaging (MRI)

T1-weighted structural MRI scans were preprocessed using SPM12. Native MRI scans were registered into stereotaxic space by applying rigid-body transformations. The registered images were segmented into gray matter (GM), white matter (WM), and CSF tissue probability maps with priori tissue maps as reference through a unified segmentation algorithm. The density map was provided by the International Consortium for Brain Mapping (ICBM), which provided the probability distribution of GM, WM, and CSF in standard spatial 2 mm × 2 mm × 2 mm. GM images were spatially normalized to the standard MNI space. Finally, the normalized GM images were smoothed with 8 mm × 8 mm × 8 mm FWHM to ensure the same resolution as preprocessed PET images. For MRI and PET image fusion, the smoothed GM image was also normalized to the range of 0 to 255.

#### MRI and PET Image Fusion

Medical image fusion is the process of registering and combining multiple images from single or multiple imaging modalities to improve imaging quality. Additional information obtained from fused images can be used to more accurately locate abnormalities to increase clinical applicability of medical images for the purpose of diagnosis and assessment of medical problems (James and Dasarathy, 2014). 3D discrete wavelet transform (DWT) was used for image fusion of PET and MRI to combine the spatial and frequency characteristics of the two modalities (Vallières et al., 2015). After data pre-processing, MRI and PET had the same resolution. We applied the 3D DWT to the MRI and PET volumes up to one decomposition level using wavelet basis function symlet8. We then averaged the spatially corresponding wavelet coefficients of all PET and MRI sub-bands to obtain a single set of fused wavelet coefficients. Finally, we applied the 3D inverse DWT to the set of fused wavelet coefficients to obtain a fused MRI/PET volume. We chose the wavelet basis function symlet8 based on previous research showing that this algorithm produced fused textures with the best predictive value (Carrier-Vallières, 2013).

### Identify MCI Conversion-Related ROIs

In this study, we focused on identification of MCI conversion-related regions-of-interest (ROIs). To control the sampling variability, we used the stratified sampling method to randomly select a subset of 81 MCI-c and 82 MCI-nc closely matched in age and sex. Table 1 provides the information of demographic and statistics of clinical assessments at time of data collection for the subset subjects. After image fusion, we performed a voxel-wise two-sample *t*-test between MCI-c and MCI-nc from the subset subjects using SPM12. This analysis compared differences in normalized volumes by applying proportional scaling to minimize the effects of inter-subject variability in global volumes. A significance threshold based on spatial extent using a cluster probability of a false discovery rate (FDR) corrected *P* ≤ 0.01 and spatial extent >50 voxels was applied to the effects of interest, and surviving voxels were retained for further analyses. Significantly different brain regions were localized using the software xjView9.6^2^. These regions were treated as ROIs in subsequent studies.

### Radiomics Analysis

Radiomics analysis was based on a published radiomics tool developed^3^ by Vallières et al. (2015). In this tool, a large number of texture features extraction and wavelet band-pass filtering algorithms were implemented. The extracted feature were previously demonstrated to be useful in prediction and diagnosis of AD (Pai et al., 2012; Sørensen et al., 2013; Hwang et al., 2016; Dolph et al., 2017). Wavelet band-pass filtering was used to decompose the ROIs of each image into different wavelet domains. We applied different weights to bandpass sub-bands (LHL, LHH, LLH, HLL, HHL, and HLH) of the ROIs, compared to low- and high-frequency sub-bands (LLL and HHH) in the wavelet domain. The ratio of the weight was defined by *R* and the values of *R* were 1/2, 2/3, 1(no wavelet filtering), and 3/2. Subsequently, in each wavelet domain (*R* = 1/2, 2/3, 1, and 3/2), a total of 43 texture features were extracted using 3D analysis for each individual: 3 histogram-based textures, 9 texture features from the Gray-Level Co-occurrence Matrix (GLCM), 13 texture features from the Gray-Level Run-Length Matrix (GLRLM), 13 texture features from the Gray-Level Size Zone Matrix (GLSZM), and 5 texture features from the Neighborhood Gray-Tone Difference Matrix (NGTDM) (Supplementary Table S1 provides a list of radiomic texture features). The details of these procedures were previously described (Vallières et al., 2015, 2017; Zhou et al., 2017). Finally, a total of 172 radiomic features were extracted for each individual.

### Cox Model Analysis

For each individual, the baseline time was the time the imaging data was collected, and the endpoint was the time of AD diagnosis for MCI-c or the last follow-up time point for MCI-nc. None of the 131 McI-c were censored for reasons such as missing interviews and 132 MCI-nc without conversion within 3 years, the censoring rate was equal to 50.2%. Cox proportional hazards regression models were calculated in R^4^ employing the ‘glmnet’ and ‘survival’ packages (Friedman et al., 2010; Simon et al., 2011; Therneau and Grambsch, 2013). Given that the sampling variability that comes with grouping the training dataset and test dataset, the randomized cross validation method was used to randomly partition into 70% training dataset and 30% test dataset with multiple times (500 times). In each simple random sampling, L1-penalized Cox model based on the least absolute shrinkage and selection operator (LASSO) was trained and the top features were selected from the training dataset. Regularization parameters were selected by 10-fold cross-validation from the training dataset. The finalized model was built with these selected parameters. The prediction accuracy of the finalized model in the training dataset was evaluated by Harrell’s Consistency C (C-index). We used an untouched test dataset to rigorously evaluate the performance of our finalized model. To validate the finalized model, it was independently applied to the test dataset by calculating the prognostic index (PI) for each individual (Royston and Altman, 2013). Here, the PI is the sum of the product of the regression coefficients β_i_ and predictor variables *x*_i_ (with *i* being the index for the order of predictors in the model), as follows: PI = β_1_*x*_1_+...+β_i_*x*_i_. Based on the value of PI, the prediction accuracy could be evaluated by C-index in the test dataset (van Houwelingen, 2000). To check whether the relative risks of the finalized Cox model were correctly specified, we performed a conventional Cox regression in the test dataset with PI as single covariate to calculate the new regression coefficient (in this paper, the term ‘new regression coefficient’ was named as ‘relative risk stability’). We used the principles as following: the model relative risk can be validated by checking if this regression coefficient is equal to 1; if the regression coefficient is equal to 1, the relative risk model is valid; if regression coefficient is not equal to 1, there is a need for calibration (van Houwelingen, 2000). The random partitioning process was repeated 500 times. Then we calculated the mean and (2.5, 97.25) percentiles of the corresponding indicators based on the 500 experimental results.

### Comparative Experiment

To evaluate the effectiveness of our proposed method, we used same analysis frameworks as fused-modality images for MRI or PET images, respectively, including: (1) identification of MCI conversion-related ROIs based on MRI or PET images; (2) a total of 172 radiomic features were extracted in the corresponding ROIs for each individual; (3) the corresponding L1-penalized Cox model was constructed and top radiomic features were selected. As results, we examined five Cox models with: (1) clinical, (2) PET imaging, (3) MRI imaging, (4) fused MRI/PET imaging, and (5) combined (clinical variables combined with radiomic features for fused MRI/PET imaging) models. For the clinical model, sex, age, MMSE, and APOE ε4 genotype (positive or negative for the presence of at least one ε4 allele) as predictors; for the three imaging models, radiomic features were considered as the predictors; for the combined model, the predictors included both clinical variables (sex, age, MMSE, and APOE ε4 genotype) and radiomic features derived from fused MRI/PET imaging. In above five models, Cox models were all executed with L1 penalty. Each Cox model was independently applied to test dataset to validate the performance of each model. In this process, the randomized cross validation method was also applied in 500 times. For each randomized cross validation, differences of the evaluation indicators (C-index and relative risk stability) between each pairs of five models were calculated. Finally, the mean difference and (2.5, 97.25) percentiles for the corresponding indicators were calculated among different models. In addition, because ROIs identified in this study was based on a subset (163 subjects) whereas the randomized cross validation of Cox models were based on all 263 subjects, it may cause bias of evaluation indicators. To evaluate whether this bias is huge, we also constructed a new combined model throughout the same analysis procedures with the rest 100 subjects not used for ROIs selection and calculated evaluation indicators (C-index and relative risk stability).

## Results

### MRI/PET Image Fusion

**FIGURE 2:**
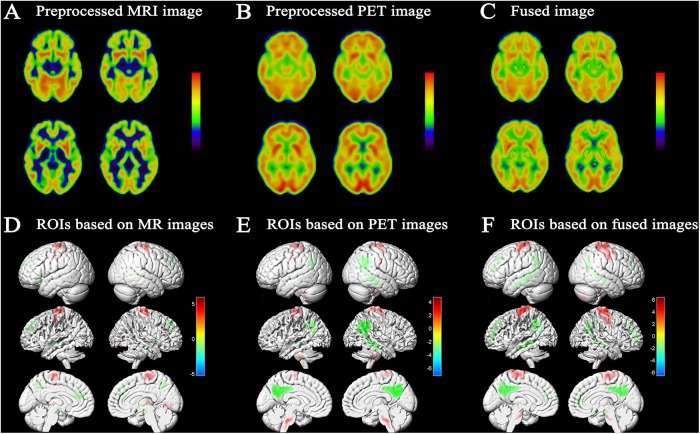
Results of image fusion and MCI conversion-related ROIs. Example of fusion **(C)** of a T1-weighted structural MRI scan **(A)** and an FDG-PET scan **(B)**; results were generated using xjView9.6 Slice Viewer. **(D)** Projection map of the voxel-wise two-sample *t*-test of GM images conducted to assess differences between MCI-c and MCI-nc. Relative reduced GM volume in MCI-c relative to MCI-nc was represented by ‘cool’ colors; relative increased GM volume in MCI-c relative to MCI-nc was represented by ‘hot’ colors (*p* < 0.01 FDR corrected, extent threshold ≥50 voxels). **(E)** Projection map of metabolic difference in MCI-c relative to MCI-nc using PET images. Relative reduced glucose metabolism was represented by ‘cool’ colors; Relative hypermetabolism was depicted by ‘hot’ colors (*p* < 0.01 FDR corrected, extent threshold ≥50 voxels). **(F)** Projection map of volume difference in MCI-c relative to MCI-nc using fused MRI/PET images. Relative reduced volume was represented by ‘cool’ colors; relative increased volume was represented by ‘hot’ colors (*p* < 0.01 FDR corrected, extent threshold ≥50 voxels).

Fusion of MRI and PET volumes was performed by a wavelet-based fusion method. Before image fusion, the preprocessed MRI and PET images were spatially normalized to the standard MNI space and converted to the same resolution. Issues such as the loss of details in image fusion caused by inconsistent image resolution were thus avoided. Figure 2C shows an example fusion of MRI (Figure 2A) and PET (Figure 2B) images. Visually, the fused image effectively preserved the edges, textures, and anatomy of multiple images. The resulting fused image was characterized as “good” in terms of features from both images that improve the quality of the imaging.

### MCI Conversion-Related ROIs

A voxel-wise two-sample *t*-test statistical model was used to identify MCI conversion-related ROIs in each imaging modality. Figure 2F shows the projection map of volume difference in MCI-c relative to MCI-nc using fused MRI/PET images. As expected, statistical analysis comparing fused images was able to capture most relevant regions. In MCI-c versus MCI-nc, relative reduced volumes were mainly localized to the temporoparietal cortex and the parahippocampal gyrus, and extended to the precuneus/posterior cingulate cortex. Regions of relative preservation included the frontal and parietal cortices, bilateral brainstem, and the precentral gyrus. Supplementary Table S4 details differences observed in this analysis.

Figure 2D shows SPM results based on MRI images in MCI-c versus MCI-nc. These brain regions, summarized in Supplementary Table S2, mainly comprised 11 clusters that were related to conversion time. Relevant regions were characterized mainly by reductions in the bilateral hippocampus, parahippocampal gyrus, inferior frontal gyrus, anterior cingulate, and a small area in the temporal lobe. Regions of relative preservation included the precentral gyrus, frontal lobe, occipital lobe, and the right thalamus.

Figure 2E shows the projection map of metabolic differences in MCI-c relative to MCI-nc based on PET images. In MCI-c versus MCI-nc, an overall reduction in glucose metabolism was observed in the temporoparietal cortex, extending to the precuneus/posterior cingulate cortex. Relative increases (most likely corresponding to regions with preserved metabolic activity) were detected in frontal and parietal cortices, cerebellum, bilateral brainstem, and precentral gyrus. Supplementary Table S3 summarizes metabolic differences between MCI-c and MCI-nc.

### Cox Models

Radiomics analysis was used to extract a total of 172 high-order quantitative features from the corresponding MCI conversion-related ROIs for each individual in each image modality (MRI/PET/fused). We used the LASSO algorithm to penalize the Cox model. The optimal regularization parameters were determined by 10-fold cross-validation on the training dataset. Features with the corresponding regression coefficients not equal to zero under the optimal regularization parameter were the features that were ultimately selected. Table 2 reported the top quantitative features selected by the corresponding Cox model in the randomized cross validations in 500 times. For the clinical model, APOE and MMSE were selected multiple times as predictors associated with associated with MCI conversion. For the Cox model constructed from MRI images, the selected quantitative features included complexity, entropy, high-gray-level zone emphasis (HGZE), busyness and coarseness. For the Cox model constructed from PET images, the selected top five quantitative features included variance, small zone emphasis (SZE), correlation, small zone high-gray-level emphasis (SZHGE) and AutoCorrelation. The predictive variables for constructing the combined model were derived from the clinical features combined with the radiomic features extracted from the fused MRI/PET imaging. The selected top quantitative features in the combined model and the Cox model constructed from fused MRI/PET images included variance, large zone low-gray-level emphasis (LZLGE), and zone-size non-uniformity (ZSN). MMSE and APOE had also been selected many times in the combined model.

**Table 2:** The top quantitative features of each Cox model selected by randomized cross validations in 500 times.

			Imaging model					
		
MRI			PET			Fused		
**Name**	***R***	**Times**	**Name**	***R***	**Times**	**Name**	***R***	**Times**

Complexity	3/2	494	Variance	1/2	475	Variance	2/3	450
Entropy	1/2	391	SZE	1/2	409	LRLGE	1/2	432
HGZE	1/2	321	Correlation	1/2	401	Strength	3/2	386
Busyness	1	269	SZHGE	1	397	ZSN	3/2	377
Coarseness	3/2	190	AutoCorrelation	3/2	253	LZLGE	1/2	364

**Clinical model**				**Combined model**				
	
**Name**	***R***		**Times**	**Name**		***R***		**Times**

MMSE	–		500	MMSE		–		500
APOE	–		498	APOE		–		473
Sex	–		299	Variance		2/3		446
Age	–		256	LZLGE		1/2		423
				ZSN		3/2		377


*Name, feature name; R, weights to bandpass sub-bands in wavelet filtering; times, the number of times corresponding feature was selected from randomized cross validations in 500 times; HGZE, high-gray-level zone emphasis; SZE, small zone emphasis; SZHGE, small zone high-gray-level emphasis; LRLGE, long-run Low-gray-level emphasis; ZSN, zone-size non-uniformity; LZLGE, large zone low-gray-level emphasis*.

**FIGURE 3:**
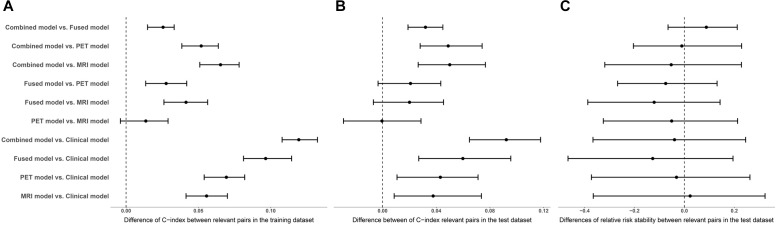
The medians and interquartile ranges of differences in performance assessment indicators between relevant pairs. **(A)** Comparison of C-index differences between relevant pairs in the training dataset. **(B)** Comparison of C-index differences between relevant pairs in the test dataset. **(C)** Comparison of differences of relative risk stability between relevant pairs in the test dataset.

The performance of the corresponding L1-penalized Cox model based on the selected top features was evaluated on the training dataset and the test dataset, respectively. First, we examined the following model using training dataset: (1) clinical model, (2) MRI model, (3) PET model, (4) fusion-modality model and (5) a combined Cox model to investigate a possible additive prognostic value of the clinical model combined with the fusion-modality model. C-index was used to evaluate the prediction accuracy of corresponding Cox model. The average C-index of each model was compared in the randomized cross validations. Table 3 listed the prediction performance comparisons for different models. Figure 3 showed the comparison of performance differences between different Cox regression models. The results showed that the prediction performances of the imaging model (MRI/PET/fused) and the combined model were better than the clinical model, and the differences in relative risk stability amongst models were not obvious. In Table 3, the imaging model (MRI model C-index = 0.7627; PET model C-index = 0.7755; fusion-modality model C-index = 0.8039) showed higher accuracy than the clinical model (C-index = 0.7066), which verified the predictive value of radiomic features from images. The fusion-modality model (C-index = 0.8039) had higher prediction accuracy than the MRI model (C-index = 0.7627) and the PET model (C-index = 0.7755). The PET model (C-index = 0.7755) was slightly better than the MRI model (C-index = 0.7627). Moreover, the combined model significantly increased the prognostic value (clinical model C-index = 0.7066; combined model C-index = 0.8268). A comparison of all models indicated that the combined model was the best model. Supplementary Table S5 listed the performance differences between relevant pairs. Second, Cox models were validated on the test dataset, and obtained PIs were used for survival analysis. Similar to the training dataset, the imaging model (MRI model C-index = 0.7330; PET model C-index = 0.7331; fusion-modality model C-index = 0.7531) had a higher C-index than the clinical model (C-index = 0.6920). The fusion model (C-index = 0.7531) had higher prediction accuracy than the MRI model (C-index = 0.7330) and the PET model (C-index = 0.7331) was also found in the test dataset. The MRI model (C-index = 0.7330) and the PET model (C-index = 0.7331) were very close in prediction performance in the test dataset. The increase of the prognostic value of the clinical model, when radiomic features for fused MRI/PET images were added as predictors to yield the combined model, was verified in the test dataset (clinical model C-index = 0.6920; combined model C-index = 0.7838). The combined model was better than the prediction performance of all other models. Third, we checked whether the relative risk of each Cox model was correctly specified. The obtained PIs as single covariate were used to perform a conventional Cox regression to obtain new regression coefficient. The closer the value of regression coefficient was to 1, the better the performance of the Cox model. In the combined model, fusion-modality model and PET model, the regression coefficients turned out to be 1.1854, 1.1122, and 1.1839, respectively; which were slightly larger than 1. Such results indicated that the relative risks of these models were correctly specified, especially in the fusion-modality model. In the clinical model and MRI model, the regression coefficients were 1.2597 and 1.2323, respectively. The regression coefficients indicated that the performance of the imaging model (MRI/PET/fused) and the combined model were better than the clinical model. Finally, the combined model based on the 100 subjects not used for ROIs selection performed similar to the combined model based on all subjects in the test dataset (0.7853 vs. 0.7930 in C-index). This meant that the bias resulted from ROIs was slight in our method.

**Table 3:** Performance evaluation of Cox regression models in the randomized cross validations.

		Harrell’s C Training dataset	Harrell’s C Test dataset	Relative risk stability Test dataset
Clinical model		***0.7066*** (0.6723, 0.7391)	***0.6920*** (0.6087, 0.7731)	***1.2597*** (0.5779, 2.3207)
Imaging model	MRI	***0.7627*** (0.7330, 0.7992)	***0.7330*** (0.6590, 0.7968)	***1.2323*** (0.6384, 1.9532)
	PET	***0.7755*** (0.7382, 0.8114)	***0.7331*** (0.6623, 0.8009)	***1.1839*** (0.6953, 1.8686)
	fused	***0.8039*** (0.7680, 0.8443)	***0.7531*** (0.6815, 0.8224)	***1.1122*** (0.6109, 1.7483)
Combined model		***0.8268*** (0.7962, 0.8562)	***0.7838*** (0.7156, 0.8490)	***1.1854*** (0.6902, 1.9057)
Combined model (100 subjects)		***0.8431*** (0.8233, 0.8598)	***0.7930*** (0.7212, 0.8193)	***1.2827*** (0.9143, 1.4893)


*C-index: Harrell’s Consistency C, used to evaluate the prediction accuracy of the Cox model. Relative risk stability: assessing the validity and value of the finalized model, we validated the model through ‘calibration’ by performing a conventional Cox regression in the test dataset with prognostic index (PI) as single covariate to calculate the new regression coefficient (van Houwelingen, 2000). Relative risk stability in the table indicates the new regression coefficient. The closer the value of relative risk stability is to 1, the better the performance of the Cox model. Combined model (100 subjects): the model was constructed using 100 subjects not used for ROIs selection. The value of the bold italic represents the average of the corresponding metrics in randomized cross validations. Parentheses represent (2.5, 97.25) percentiles*.

## Discussion

In this study, we applied radiomics analysis methods to extract radiomic features in MCI conversion-related ROIs. Then, multivariate Cox proportional hazard regression models were generated to evaluate the influence of radiomic predictors of interest on time to onset of AD dementia among individuals with MCI. Our findings indicated that a combination of radiomic analysis and Cox model analyses could be used for MCI conversion prediction.

### MCI Conversion-Related Topography

In our study, regions of structural atrophy and functional metabolic abnormalities associated with MCI conversion as defined by SPM analysis were also reported in previous studies (Jack et al., 2000; Habeck et al., 2008; Desikan et al., 2010; De Oliveira et al., 2011; Devanand et al., 2012; Prestia et al., 2013a; Zeifman et al., 2015; Mattis et al., 2016; Liu et al., 2017). Based on MRI, MCI conversion-related ROIs were characterized mainly by reductions in the bilateral hippocampus, parahippocampal gyrus, inferior frontal gyrus, anterior cingulate, and a small area in the temporal lobe; regions of relative preservation included the precentral gyrus, frontal lobe, occipital lobe, and the right thalamus. The latter most likely corresponds to preserved GM volume, given that proportional scaling by global uptake was performed. The main brain regions related to MCI conversion identified in the current study were consistent with those reported in the literature Predicting (Jack et al., 2000; Desikan et al., 2010; Devanand et al., 2012; Prestia et al., 2013a; Zeifman et al., 2015; Korolev et al., 2016; Liu et al., 2017). Previous reports showed that GM atrophy was associated with increased risk of progression to AD (Tapiola et al., 2008; Devanand et al., 2012; Prestia et al., 2013a; Zeifman et al., 2015). Jack et al. reported that hippocampal atrophy correlated with AD-related changes in clinical status (Jack et al., 2000). Zeifman et al. (2015) demonstrated that three brain regions were significantly associated with time to AD: the mesial temporal lobe, the anterior hippocampus extending into the amygdala, and the posterior cingulate gyrus. In addition, Desikan et al. (2010) found that automated MRI measurement of the medial temporal cortex accurately and reliably predicted time to AD progression for individuals with MCI. Using PET, we found that conversion to AD is characterized by significant metabolic decrease in the temporoparietal cortex, extending to the precuneus/posterior cingulate cortex, while frontal and parietal cortices, cerebellum, bilateral brainstem, and precentral gyrus showed increased metabolic activity. These results were consistent with previously published hypo- and hypermetabolic regions identified in AD (Habeck et al., 2008; Teune et al., 2014; Mattis et al., 2016; Liu et al., 2017). We also analyzed the same groups of subjects with SPM t-test using fused MRI/PET imaging, which resulted in overlapping regions of reduction and preservation of GM volume with MRI imaging alone or hypo- and hypermetabolism with PET imaging alone. These results showed that the fused image could retain additional information from each single modality image (James and Dasarathy, 2014).

### Radiomic Features

Recently, radiomic analysis has been successfully used to identify imaging biomarkers for diseases besides cancer, especially AD (De Oliveira et al., 2011; Zhang et al., 2012; Anandh et al., 2015; Chincarini et al., 2016; Chaddad et al., 2017; Sun et al., 2017; Feng et al., 2018). Feng et al. (2018) identified hippocampal radiomic features as potential biomarkers for clinical evaluation of AD, indicating that texture may serve as a prognostic neuroimaging biomarker for early cognitive impairment (Sørensen et al., 2016). A texture analysis study found texture differences in the corpus callosum and thalamus in MRI images of patients with mild AD and aMCI. Zhang et al. (2012) demonstrated that 3D texture could be used as a possible diagnostic marker of AD using T1-weighted MR images.

Radiomics mainly includes the following steps: (1) acquisition of high-quality images; (2) identification of regions of interest, which are segmented with operator edits, then rendered in 3D; (3) extraction of quantitative features from these rendered volumes for analysis with other data (such as clinical and genomic data) to develop diagnostic, predictive, or prognostic models for outcomes of interest (Gillies et al., 2015). Since a key step in implementation of radiomics involves segmentation of lesions (Kumar et al., 2012; Gillies et al., 2015), it is necessary to use statistical analyses to determine ROIs. After SPM analysis, the ROIs mask was obtained and features were extracted from these ROIs. In total, 172 radiomic features were extracted for each subject in each image modality (MRI/PET/fused), including intensity, texture, and wavelet features. Recent radiomics studies evaluated shape features (Kumar et al., 2012; Aerts et al., 2014; Gillies et al., 2015; Vallières et al., 2015; Huang et al., 2016; Zhou et al., 2017) because the target areas, such as tumor areas, were segmented manually. However, these shapes were unified by the ROIs mask in this study. Moreover, individual differences in tumors were much greater than differences in normal brain tissue. For brain MRI studies, preprocessing steps generally include spatial registration and normalization. In this study, we followed this preprocessing process, which further reduced the effects of shape differences. Therefore, our feature set did not contain shape features.

The top quantitative features in the MRI imaging modality, which included entropy, complexity and coarseness, etc., exhibited the most significant alterations associated with MCI conversion. In probability theory and statistics, energy derived from GLCM is one of the histogram parameters that are concerned with information content of an image and describes the complexity of the image (Haralick et al., 1973). Complexity and coarseness derived from GLRLM could reflect the degree of association between different pixels in the same brain region (Amadasun and King, 1989). Significant differences in the above features indicate that brain structural impairments in MCI-c might result in complicated and altered distributions of voxel values within the MCI conversion-related regions. This inference was supported by previous studies (Feng et al., 2018). In the PET model, fusion-modality model and combined model, the corresponding top quantitative features were selected, respectively. These features captured both the local and more global texture patterns associated with MCI conversion in the training dataset and could re-predict the risk of MCI conversion in the test dataset. These quantitative image features are generally difficult to spot by manual inspection, but computerized methods can effectively identify such features. These features were most highly correlated with the time of onset and outcome of MCI conversion in each imaging modality, demonstrating the value of high-order quantitative image features for clinical diagnosis and prognosis.

### Cox Proportional Hazards Regression Model

In Cox model analysis, the clinical Cox model was constructed to include only clinical variables. The results showed that APOE ε4 gene can be used as risk predictors to predict MCI conversion. Many studies have suggested that the APOE ε4 gene is a significant genetic risk factor for sporadic and late onset familial AD (Furney et al., 2011; Trachtenberg et al., 2012; Murphy et al., 2013; Risacher et al., 2015). Liu et al. (2017) demonstrated that individuals with MCI who had a positive APOE ε4 status at baseline were at higher risk for converting to AD within 3 years. In our study, the imaging Cox model with radiomic features was comprehensive, and provided significant enhancement in prognosis of conversion compared to the clinical model, especially the fusion-modality Cox model. Harrell’s C and relative risk stability were used to evaluate the performance of the prediction. The Cox model constructed based on top radiomic features derived from the fused MRI/PET images resulted in a higher Harrell’s C and a more stable relative risk. This finding indicated that radiomic features from fused image modality were with more predictive value than single-modality for estimating risk associated with MCI progression. It may be because the Cox model of the fusion-modality incorporated complementary information between both PET and MRI images. Other studies have also demonstrated that Cox models based on multimodal images exhibit superior predictive and diagnostic value compared to single modality models (Jack et al., 2010; Chen et al., 2011; Dickerson and Wolk, 2013; Liu et al., 2017). In our study, the combination of imaging and clinical variables provided the best predictive data, similar to a study by Liu et al. (2017). This suggests that inclusion of multiple types of risk factors increases the predictive power of the Cox model for estimating the risk associated with MCI progression (Chen et al., 2016; Korolev et al., 2016). Furthermore, radiomics analysis could be applied to a new patient on a single-case basis. The benefit of the current study is the ability to combine significant radiomic features with clinical variables to obtain a quantifiable prognostic index for conversion of MCI to AD on an individual basis, which may be a particularly attractive approach for single-patients predictions and very important for clinical purposes.

## Limitations and Further Considerations

Although use of radiomic features as predictive factors in pre-diagnosis of AD has been explored in the present study, some limitations exist. The first limitation of this study is the relatively short follow-up period for participants with MCI. Individuals with MCI were followed for 3 years, consistent with previous studies of MCI. Furthermore, a proportion of MCI images did not have baseline data for both MRI and FDG PET imaging available at the time of study. Future studies will consider multimodal data from a larger number of MCI-c subjects whose long-term visits may help improve performance of our predictive model (Chen et al., 2016). Second, considering the small number of experimental subjects, we did not use another independent subject to validate MCI conversion-related ROIs, besides a subset of our experimental subjects. Finally, there was a smoothing step in preprocessing, which may affect calculation of features and definition of ROIs. This is a routine step in other AD studies, so we followed this principle (Devanand et al., 2007; Schneider et al., 2009; Gomar et al., 2011; Vos et al., 2012; Prestia et al., 2013b; Teipel et al., 2013; Duan et al., 2017). In future research, the target region should be extracted separately for each person using a more accurate method, such as manual segmentation, in a manner similar to oncological radiomics studies (Gillies et al., 2015).

## Conclusion

In summary, the findings of the present investigation suggest that a combination of radiomic analysis and Cox model analyses could be successfully used in survival data analysis to predict MCI to AD progression. Furthermore, our findings indicated that top risk factors derived from fused MRI/PET neuroimaging, together with clinical variables, can effectively predict MCI conversion. The simplicity of acquisition of radiomic features and high-throughput nature demonstrate the power of this technique for development of personalized precision medicine for the population affected by AD.

## Ethics Statement

This study was carried out in accordance with the recommendations of Good Clinical Practice guidelines, US 21CFR Part 50 – Protection of Human Subjects, and Part 56 – Institutional Review Boards (IRBs)/Research Ethics Boards (REBs). The protocol was approved by Institutional Review Boards (IRBs)/Research Ethics Boards (REBs).

## Author Contributions

JJ conceived and designed the experiments, analyzed and interpreted the data, and wrote the manuscript. HZhou, JL, MW, and HZhang performed the experiments, and wrote the manuscript. CZ analyzed and interpreted the data and wrote the manuscript. The Alzheimer’s Disease Neuroimaging Initiative contributed reagents, materials, and data.

## Conflict of Interest Statement

The authors declare that the research was conducted in the absence of any commercial or financial relationships that could be construed as a potential conflict of interest.

## Footnotes

1 http://adni.loni.usc.edu/

2 http://www.alivelearn.net/xjview

3 https://github.com/mvallieres/radiomics

4 http://www.R-project.org/
