# Supplementary material for: Dual-Model Radiomic Biomarkers Predict Development of Mild Cognitive Impairment Progression to Alzheimer’s Disease
Source: Front Neurosci. 2019 Jan 11;12:1045. doi: 10.3389/fnins.2018.01045 (PMC6338093; doi:10.3389/fnins.2018.01045)
Supplement: Supplementary file 1 [file Table_1.DOCX]

**Supplementary material**

Table S1 Details of radiomic texture features.

| Texture matrices | References | Feature Name |
| --- | --- | --- |
| Global |  | Variance  Skewness  Kurtosis |
| Gray-level co-occurrence matrix  (GLCM) | (Haralick et al., 1973) | Energy  Contrast  Correlation  Homogeneity  Variance  Sum Average  Entropy  AutoCorrelation  Dissimilarity |
| Gray-level run-length matrix  (GLRLM) | (Galloway, 1974) | Short-run emphasis (SRE)  Long-run emphasis (LRE)  Gray-level nonuniformity (GLN)  Run-length nonuniformity (RLN)  Run percentage (RP) |
|  | (Chu et al., 1990) | Low-gray-level run emphasis (LGRE)  High-gray-level run emphasis (HGRE) |
|  | (Dasarathy and Holder, 1991) | Short-run low-gray-level emphasis (SRLGE)  Short-run high gray-level emphasis (SRHGE)  Long-run Low-gray-level emphasis (LRLGE)  Long-run high-gray-level emphasis (LRHGE) |
|  | (Thibault et al., 2009) | Gray-level variance (GLV)  Run-length variance (RLV) |
| Gray-level size  zone matrix  (GLSZM) | (Galloway, 1974;Thibault et al., 2009) | Small zone emphasis (SZE)  Large zone emphasis (LZE)  Gray-level nonuniformity (GLN)  Zone-size nonuniformity (ZSN)  Zone percentage (ZP) |
|  | (Chu et al., 1990;Thibault et al., 2009) | Low-gray-level zone emphasis (LGZE)  High-gray-level zone emphasis (HGZE) |
|  | (Dasarathy and Holder, 1991;Thibault et al., 2009) | Small zone low-gray-level emphasis (SZLGE)  Small zone high-gray-level emphasis(SZHGE)  Large zone low-gray-level emphasis (LZLGE)  Large zone high-gray-level emphasis(LZHGE) |
|  | (Thibault et al., 2009) | Gray-level variance (GLV)  Zone-size variance (ZSV) |
| Neighborhood gray-tone difference matrix  (NGTDM) | (Amadasun and King, 1989) | Coarseness  Contrast  Busyness  Complexity  Strength |

Table S2 Regions-of-interest with significant differences based on MRI scans between 81 MCI-c patients and 82 MCI-nc patients in training set.

| Cluster | Region | BA | Laterality | KE | Peak voxel | | | |
| --- | --- | --- | --- | --- | --- | --- | --- | --- |
|  |  |  |  |  | T | x | y | z |
| Relative increased gray matter volume in MCI-c | | | | | | | | |
| 1 | Precentral_R  Paracentral_Lobule_R  Supp_Motor_Area_R  Postcentral_R | 6 | Right | 865 | 5.90 | 32 | -16 | 68 |
| 2 | Precentral Gyrus  Postcentral_L | 6,4 | Left | 862 | 5.63 | -16 | -14 | 48 |
| 3 | Occipital Lobe  Lingual_R | 18,19 | Right | 253 | 4.62 | 16 | -60 | -6 |
| 4 | Thalamus_R |  | Right | 142 | 4.41 | 18 | -20 | 4 |
| Relative reduced gray matter volume in MCI-c | | | | | | | | |
| 1 | Hippocampus_R/  Parahippocampa Gyrus |  | Right | 303 | -4.69 | 20 | -12 | -8 |
| 2 | Superior Frontal Gyrus | 9,10 | Right | 275 | -5.16 | 20 | 38 | 40 |
| 3 | Hippocampus_L  Parahippocampa Gyrus |  | Left | 271 | -4.84 | -20 | -16 | -8 |
| 4 | Superior Frontal Gyrus  Frontal_Mid_L |  | Left | 248 | -5.03 | -18 | 54 | 26 |
| 5 | Anterior Cingulate  Medial Frontal Gyrus | 32,9,24 | Left | 221 | -4.40 | -8 | 42 | 14 |
| 6 | Precuneus_R  Precuneus_L | 7 | Right | 147 | -4.27 | 2 | -64 | 54 |
| 7 | Inferior Frontal Gyrus  Insula_L | 47 | Left | 136 | -4.27 | -38 | 24 | 0 |
| 8 | Temporal Lobe | 21 | Right | 109 | -4.24 | 58 | -30 | -2 |

Voxel size = 2 mm$\times$2 mm$\times$2 mm.

Key: BA, Brodmann area; KE, cluster extent.

Table S3 Regions-of-interest with significant differences based on PET scans between 81 MCI-c patients and 82 MCI-nc patients in training set.

| Cluster | Region | BA | Laterality | KE | Peak voxel | | | |
| --- | --- | --- | --- | --- | --- | --- | --- | --- |
|  |  |  |  |  | T | x | y | z |
| Increased metabolism in MCI-c | | | | | | | | |
| 1 | Precentral_R  Parietal Lobe | 6,3 | Right | 619 | 4.38 | 12 | -34 | 72 |
| 2 | Precentral_L  Parietal Lobe | 6 | Left | 559 | 4.58 | -28 | -18 | 72 |
| 3 | Left Brainstem  Right Brainstem |  | Left  Right | 420 | 4.62 | -4 | -36 | -40 |
| 4 | Cerebelum_8_L |  | Left | 148 | 3.87 | -22 | -50 | -54 |
| Decreased metabolisma in MCI-c | | | | | | | | |
| 1 | Parietal Lobe  Temporal Lobe  Angular_R | 40,39,21,22 | Right | 3166 | -6.08 | 48 | -54 | 36 |
| 2 | Precuneus  Parietal Lobe  Posterior Cingulate  Occipital Lobe | 7,31,7,23 | Right | 2553 | -7.05 | 4 | -62 | 30 |
| 3 | Angular_L  Temporal Lobe  Inferior Parietal Lobule | 39,40 | Left | 794 | -4.88 | -44 | -60 | 34 |
| 4 | Middle Temporal Gyrus |  | Left | 154 | -3.60 | -56 | -24 | -10 |

Voxel size = 2 mm$\times$2 mm$\times$2 mm.

Key: BA, Brodmann area; KE, cluster extent.

Table S4 Regions-of-interest with significant differences based on fused MRI/PET scans between 81 MCI-c patients and 82 MCI-nc patients in training set.

| Cluster | Region | BA | Laterality | KE | Peak voxel | | | |
| --- | --- | --- | --- | --- | --- | --- | --- | --- |
|  |  |  |  |  | T | x | y | z |
| Relative increased volume in MCI-c | | | | | | | | |
| 1 | Precentral Gyrus  Frontal Lobe  Postcentral Gyrus  Parietal Lobe | 6,4,3 | Right | 2121 | 6.31 | 26 | -18 | 70 |
| 2 | Frontal Lobe  Precentral Gyrus  Parietal Lobe  Postcentral Gyrus | 6,3 | Left | 1416 | 5.85 | -34 | -16 | 66 |
| 3 | Left Brainstem  Right Brainstem |  | Left  Right | 199 | 4.28 | -6 | -24 | -34 |
| 4 | Thalamus_R  Right Brainstem |  | Right | 139 | 5.54 | 20 | -12 | -2 |
| 5 | Superior Frontal Gyrus |  | Left | 127 | 3.95 | -16 | -6 | 78 |
| Relative reduced volume in MCI-c | | | | | | | | |
| 1 | Middle Temporal Gyrus  Parietal Lobe  Superior Temporal Gyrus  Supramarginal Gyrus | 22 | Left | 2167 | -5.47 | -58 | -38 | -2 |
| 2 | Cingulate Gyrus  Precuneus  Parietal Lobe | 31,7 | Right | 1962 | -6.46 | 2 | -32 | 38 |
| 3 | Temporal_Mid_R  Angular_R  Parietal Lobe |  | Right | 944 | -5.13 | 46 | -60 | 32 |
| 4 | Middle Frontal Gyrus  Superior Frontal Gyrus | 10,9 | Left | 678 | -5.23 | -34 | 48 | 16 |
| 5 | Middle Frontal Gyrus  Superior Frontal Gyrus |  | Right | 480 | -4.18 | 18 | 54 | 26 |
| 6 | Hippocampus_R  Parahippocampa Gyrus | 28,35 | Right | 211 | -5.34 | 22 | -10 | -12 |
| 7 | Anterior Cingulate  Medial Frontal Gyrus | 32 | Right | 148 | -4.36 | 8 | 44 | 14 |

Voxel size = 2 mm$\times$2 mm$\times$2 mm.

Key: BA, Brodmann area; KE, cluster extent.

Table S5 Comparison of performance differences between relevant pairs.

|  | △C-index  **Training dataset** | △C-index  **Test dataset** | △Relative risk stability  **Test dataset** |
| --- | --- | --- | --- |
| MRI vs. Clinical | ***0.0557***  (0.0164, 0.0997) | ***0.0376***  (-0.0470, 0.1384) | ***0.0230***  (-1.1577, 0.8857) |
| PET vs. Clinical | ***0.0694***  (0.0289, 0.1137) | ***0.0431***  (-0.0496, 0.1281) | ***-0.0318***  (-1.1453, 0.7965) |
| Fused vs. Clinical | ***0.0965***  (0.0461, 0.1469) | ***0.0598***  (-0.0286, 0.1562) | ***-0.1270***  (-1.2917, 0.7605) |
| Combined vs. Clinical | ***0.1194***  (0.0851, 0.1581) | ***0.0922***  (0.0179,0.1609) | ***-0.0398***  (-1.1311, 0.7769) |
| PET vs. MRI | ***0.0136***  (-0.0339, 0.0599) | ***-0.0005***  (-0.0807, 0.0763) | ***-0.0512***  (-0.8428, 0.7426) |
| Fused vs. MRI | ***0.0414***  (-0.0037, 0.0859) | ***0.0200***  (-0.0523, 0.0910) | ***-0.1214***  (-0.9554, 0.6834) |
| Fused vs. PET | ***0.0277***  (-0.0097, 0.0741) | ***0.0208***  (-0.0561, 0.0914) | ***-0.0749***  (-0.7569, 0. 6009) |
| Combined vs. MRI | ***0.0653***  (0.0214,0.0999) | ***0.0501***  (-0.0274, 0.1279 | ***-0.0530***  (-0.9056, 0.7034) |
| Combined vs. PET | ***0.0519***  (0.0142, 0.0830) | ***0.0490***  (-0.0157, 0.1112) | ***-0.0106***  (-0.6581, 0.6882) |
| Combined vs. Fused | ***0.0256***  (-0.0154, 0.0498) | ***0.0319***  (-0.0207, 0.0691) | ***0.0881***  (-0.4206, 0.5550) |

Note: C-index: Harrell's Consistency C, used to evaluate the prediction accuracy of Cox model.

△C-index: the differences in C-index between relevant pairs. Relative risk stability: assessing the validity and value of the the finalized model, we validated the model through ‘calibration’ by performing a conventional Cox regression in the test set with prognostic index (PI) as single covariate to calculate the new regression coefficient(van Houwelingen, 2000). Relative risk stability in the table indicates the new regression coefficient. The closer the value of relative risk stability is to 1, the better the performance of the Cox model. △Relative risk stability: the differences in relative risk stability between relevant pairs. The value of the bold italic represents the median of the corresponding metrics in the 500 randomized cross validations. Parentheses represent (2.5, 97.25) percentiles of these differences by randomized cross validations in 500 times.

**References**

Amadasun, M., and King, R. (1989). Textural features corresponding to textural properties. *IEEE Transactions Systems Man & Cybernetics* 19**,** 1264-1274.

Chu, A., Sehgal, C.M., and Greenleaf, J.F. (1990). Use of gray value distribution of run lengths for texture analysis. *Pattern Recognition Letters* 11**,** 415-419.

Dasarathy, B.V., and Holder, E.B. (1991). Image characterizations based on joint gray level—run length distributions. *Pattern Recognition Letters* 12**,** 497-502.

Galloway, M.M. (1974). Texture analysis using grey level run lengths. *Nasa Sti/recon Technical Report N* 75.

Haralick, R.M., Shanmugam, K., and Dinstein, I.H. (1973). Textural Features for Image Classification. *Systems Man & Cybernetics IEEE Transactions on* smc-3**,** 610-621.

Thibault, G., Fertil, B., Navarro, C., Pereira, S., Levy, N., Sequeira, J., and Mari, J.L. (2009). Texture Indexes and Gray Level Size Zone Matrix Application to Cell Nuclei Classification. *In Pattern Recognition and Information Processing (PRIP*.

Van Houwelingen, H.C. (2000). Validation, calibration, revision and combination of prognostic survival models. *Statistics in medicine* 19**,** 3401-3415.
